# Integrated Control of Thermal Residual Stress and Mechanical Properties by Adjusting Pulse-Wave Direct Energy Deposition

**DOI:** 10.3390/ma17215231

**Published:** 2024-10-27

**Authors:** Zhou Yan, Jia Guo, Xi Zou, Siyu Wang

**Affiliations:** 1 School of Information Engineering, Hubei University of Economics, Wuhan 430205, China; 2 Hubei Key Laboratory of Digital Finance Innovation, Hubei University of Economics, Wuhan 430205, China; 3 Hubei Internet Finance Information Engineering Technology Research Center, Hubei University of Economics, Wuhan 430205, China; 4 Mechanical and Electrical Engineering College, Hunan University of Science and Technology, Xiangtan 411201, China; 5 Graduate School of Computer and Information Sciences, Hosei University, Tokyo 184-8584, Japan; 6 Department of Automation, Shanghai Jiao Tong University, Shanghai 201100, China

**Keywords:** direct energy deposition, residual stress, temperature gradient, geometric dislocation density, mechanical property

## Abstract

Directed energy deposition with laser beam (DED-LB) components experience significant residual stress due to rapid heating and cooling cycles. Excessive residual tensile stress can lead to cracking in the deposited sample, resulting in service failure. This study utilized digital image correlation (DIC) and thermal imaging to observe the in situ temporal evolution of strain and temperature gradients across all layers of a deposited 316 L stainless steel thin wall during DED-LB. Both continuous-wave (CW) and pulsed-wave (PW) laser modes were employed. Additionally, the characteristics of thermal cracks and geometric dislocation density were examined. The results reveal that PW mode generates a lower temperature gradient, which in turn reduces thermal strain. In CW mode, the temperature–stress relationship curve of the additive manufacturing sample enters the “brittleness temperature zone”, leading to the formation of numerous hot cracks. In contrast, PW mode samples are almost free of cracks, as the metal avoids crack-sensitive regions during solidification, thereby minimizing hot crack formation. Overall, these factors collectively contribute to reduced residual stress and improved mechanical properties through the adjustment of pulsed-wave laser deposition.

## 1. Introduction

As a breakthrough in the manufacture of complex parts, additive manufacturing (AM) entirely allows product design freedom and offers for the customization of microstructure and mechanical properties [1,2]. Laser directed energy deposition (L-DED-LB), which commonly uses a continuous wave (CW) laser to melt metal powders before constructing components layer-by-layer, is one of the most promising AM processes [3,4]. During such processes, temperature gradients can reach up to 1000 K/m, and cooling rates can exceed 10^3^~10^4^ K/s [5]. In comparison to powder bed techniques, wire fed DED, such as laser based DED (DED-LB/M) and wire arc additive manufacturing (WAAM), offers better structural performance since it has fewer intrinsic faults and high productivity because of its high displacement rate.

However, DED-LB can generate a substantial amount of residual stress. Excessive residual tensile stress may lead to cracking in the deposited sample, compromising its functionality. Therefore, controlling residual stress and enhancing mechanical properties are crucial for optimizing the quality of additively manufactured metal components [6,7,8].

In general, residual stresses arise from several factors [9]: (1) Spatial Temperature Gradient: The moving heat source rapidly heats and cools both the substrate and the material used to deposit the sample, resulting in substantial temperature differentials. (2) Thermal Expansion and Contraction: Due to spatial temperature differentials and constraints from surrounding metal, thermal expansion and contraction generate significant thermal strains. (3) Plasticity and Flow Stress: As the heat source moves, stress is “instantly” redistributed to establish a new static equilibrium with each new temperature field. (4) Grain Properties and Phase Composition: The mobility of dislocations, driven primarily by thermal stress from temperature gradients, leads to an increase in geometrically necessary dislocations (GND). Additionally, phase transitions resulting from thermal history significantly influence residual stress formation during directed energy deposition (DED-LB).

The presence of tensile residual stress within deposited structures compromises their structural integrity and service performance by promoting crack initiation and propagation [10]. Therefore, accurately determining the temperature gradient and temporal thermal strains within deposited materials is essential for exploring methods to reduce thermal stress and, ultimately, residual stress.

Additionally, by correlating non-destructive testing (NDT) results with specific process parameters, it is observed that higher defect density is present in coupons with smaller heat-affected zone (HAZ) dimensions—indicative of lower energy density—which promotes crack formation due to high thermal stresses incurred during manufacturing.

Most studies focus on measuring the final deformation of substrates using coordinate measuring machines or 3D laser scanning equipment. Gao et al. [11] examined the influence of laser beam direction on substrate distortion during direct deposition. Afazov et al. [12] analyzed the maximum distortion deviation in cantilever beams fabricated by selective laser melting. However, measuring strain and deformation in real time throughout the manufacturing process remains challenging. Denlinger and Heigel [13,14] attempted to capture instantaneous deformation of a cantilevered substrate during deposition and cooling using a laser displacement sensor (LDS). Despite its utility, this method only examines a limited section of the substrate, as measuring the entire deposit’s distortion and strain in situ would be too time-consuming.

The digital image correlation (DIC) technique, initially developed by V. Ocelk et al. [15] for in situ monitoring of dynamic full-field strain during laser cladding, has proven useful for capturing transient distortion and strain in additive manufacturing. However, the relationship between thermal strain, residual stress, and temperature gradient remains insufficiently explored. Consequently, the reduction and control of residual stress in AM-deposited samples have not been optimized.

A pulse-wave (PW) laser DED-LB approach was recently introduced to enhance grain structure and prevent elemental segregation in various metals and alloys [16,17]. In PW-DED-LB, periodic laser input causes continuous variation in molten-pool shape and solidification parameters [16,17]. This periodic adjustment significantly influences the temperature gradient, thermal strain field, residual stress field, and mechanical properties, enabling manipulation of the solidification microstructure. However, the mechanisms controlling residual stress, strain, and mechanical properties during PW-DED-LB are not yet fully understood.

The primary objective of this work is to investigate the in situ temperature gradient, thermal strain field, residual stress field, and mechanical properties of samples deposited using CW and PW laser modes under constant laser power conditions. This study aims to elucidate the relationships between temperature gradient, thermal strain, and residual stress, as well as to reveal how PW laser mode reduces and homogenizes residual stress in deposited samples. The findings provide both theoretical and technical guidance for manufacturing laser additive components with minimal residual stress. Additionally, the structural properties and mechanical evolution of PW mode under various heat treatment conditions were examined, offering a comprehensive approach to controlling residual stress and mechanical properties.

## 2. Experiments and Simulation Methods

### 2.1. Experiments

**Figure 1:**
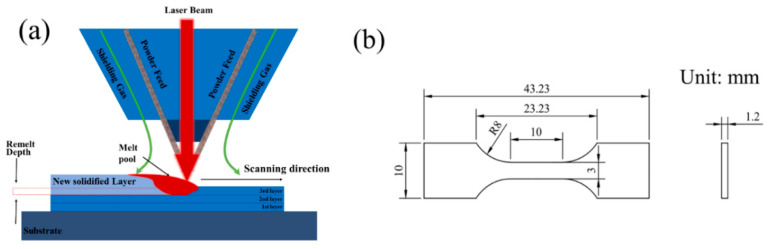
(**a**) Diagram illustrating the experimental method for the DED-LB. (**b**) Measurements of the dimensions for the tensile sample.

**Figure 2:**
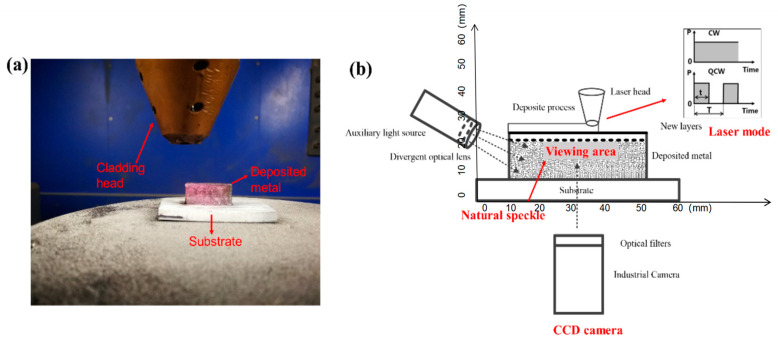
(**a**) Schematic representation of the experimental setup. (**b**) The industrial camera and thermal imager captured an in situ image of the deposited metal. The freshly added DED-LB layer of the sample appears as a metallic grey color on the top of the wall sample.

In this study, powder-feeding additive manufacturing was employed using a 316 L stainless steel substrate (60 mm × 60 mm × 10 mm) and 316 L stainless steel powder, as shown in Figure 1a. The tensile test specimen’s dimensions are illustrated in Figure 1b, and Table 1 provides material details. To monitor temperature variations, a BM_IR thermal imager was utilized, while instantaneous strain was observed with an industrial camera equipped with optical filters, enabling clear visualization of the deposition area (as shown in Figure 2) by filtering out extraneous light. The camera, with a resolution of 640 × 480 pixels, captured images at 225 milliseconds exposure and 40 fps, with strain field data analyzed using VIC-2D software. The VIC-2D system is a fully integrated solution that utilizes optimized correlation algorithms to provide non-contact, full-field, two-dimensional displacement and strain data for mechanical testing on planar specimens. In-plane displacements are measured at every point within the area of interest, and the full-field strain field is computed and displayed to easily identify strain concentrations. For the experiment, two single-wall DED-LB samples (25 mm length, 15 mm height) were fabricated on the substrate, and high-temperature black-and-white speckles were applied for transient full-field strain observation. The VIC-2D software was used to compute strain distribution. Additionally, the contour method, effective for mapping residual stress, was employed to determine residual stresses in the DED-LB sample. This method provides two-dimensional residual stress maps based on elastic superposition theory, where residual stress is relaxed by cutting and surface displacement (measured by a Coordinate Measuring Machine) is compared to a planar contour. The process involves cutting, surface measurement, and stress calculation, with detailed methodology available in references [18,19,20].

A laser power of 450 W was used in both continuous-wave (CW) and pulsed-wave (PW) laser modes to examine the effects of the laser mode on the temperature gradient and transient strain, while maintaining the same power level. As defined in Figure 2b, the PW pulse cycle time and duty ratio were set to 0.2 s (with a pulse frequency of 5 Hz) and 50%, respectively. All other conditions remained constant, with the element composition of 316 L powder and the process parameters provided in Table 1 and Table 2. Each sample consisted of six layers, with an elevation of 0.23 mm per layer, fabricated using single-channel scanning with 4 s for both heating and cooling. Additional processing parameters included a powder feeding rate of 11.5 g/min, a scanning speed of 6 mm/s, a protective argon gas flow rate of 8 L/min, and a spot diameter of 1 mm.

### 2.2. Thermo-Mechanical Calculations

**Figure 3:**
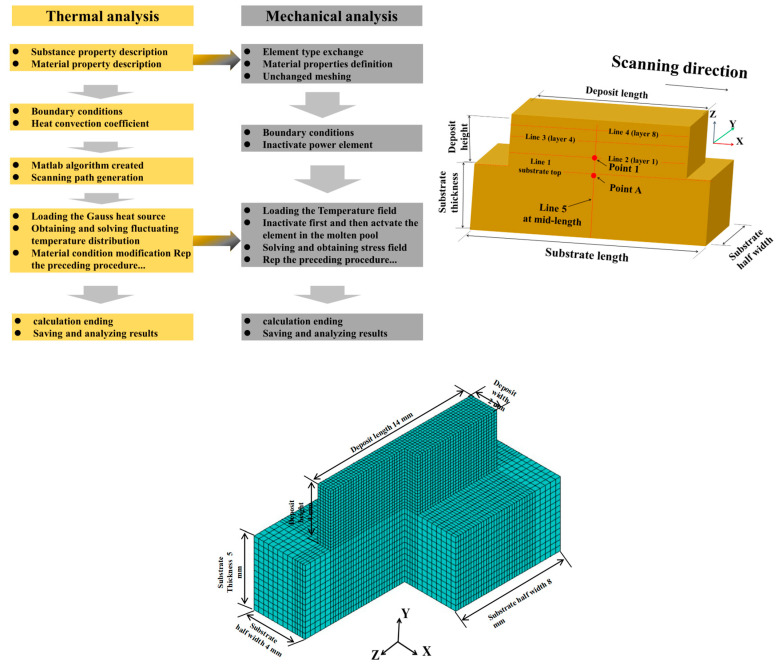
Schematic diagram of numerical simulation.

The finite element method (FEM) was used to simulate the development of the residual stress field during directed energy deposition (DED-LB) in austenitic stainless steels. Figure 3 illustrates the simulation methodology and the dimensions of the model [21]. The basic formula for transient heat conduction in the DED-LB process is listed in Equation (1):

(1) $$Q\left(x,\;t\right)-\nabla *q(x,t)=\rho C_{p}\frac{dT}{dt}$$

where *ρ* represents the density; *C_p_* represents the temperature-dependent specific heat capacity; *T* represents the temperature; *t* represents the time; *q* represents the conductive heat flux through the material.

For a laser beam, the average volumetric heat source model is shown in Equation (2):

(2) Q(x,t)=ηP/V

where *η* represents alloy laser absorption, *P* represents laser power, and *V* is the volume impacted by the heat source.

The equation for maintaining mechanical stress equilibrium may be expressed in Equation (3):

(3) ∇∗σ=0

where *σ* represents the third-order stress tensor.

The mechanical constitutive law can be represented in Equation (4):

(4) $$\sigma =C\varepsilon_{e}$$

when *C* represents the fourth-order stiffness tensor, and $\varepsilon_{e}$ is as defined in Equation (5):

(5) $$\varepsilon_{e}=\varepsilon -\varepsilon_{p}-\varepsilon_{T}$$

where $\varepsilon_{e}$ represents total strain, $\varepsilon_{p}$ represents plastic strain, and $\varepsilon_{T}$ represents thermal strain. In DED-LB, the equivalent stress ($\varepsilon_{e}$), also known as von Mises stress, is commonly used to measure residual stress [21].

## 3. Results

### 3.1. Evolution of the In Situ Temperature Gradient

**Figure 4:**
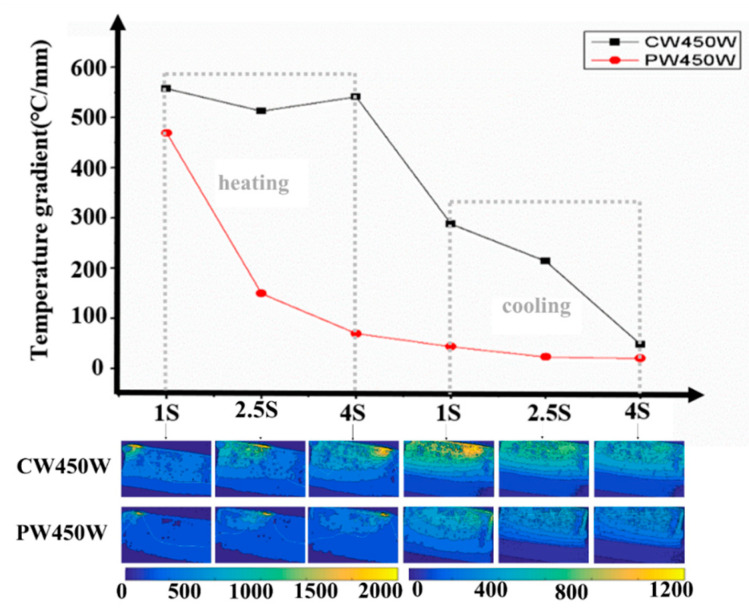
X-temperature gradient distribution with one-layer laser deposition (CW and PW mode) at Point 1.

Figure 4 presents 2-D maps of the temperature distribution and X-direction temperature gradient at Point 1 under different laser modes during single-layer laser deposition. The X-direction temperature gradient data were extracted from the temperature distribution, following the method outlined in [22]. As the laser arrives, the metal heats up, and the temperature gradient sharply increases. The temperature gradient reaches its maximum when the laser is directly over the area: 558.47 °C/mm for CW mode and 465.75 °C/mm for PW mode. As the laser moves away, the metal begins to cool, and the temperature gradient gradually decreases until it approaches 0 °C/mm.

Notably, the temperature gradient in CW mode is significantly higher than in PW mode during single-layer laser deposition. For CW mode, the temperature gradient at Point 1 remains between 500 °C/mm and 560 °C/mm throughout the heating phase, only dropping rapidly during the cooling phase. In contrast, for PW mode, the temperature gradient decreases gradually during both the heating and cooling phases of the single-layer deposition. This behavior is due to the metastable nature of the PW temperature gradient, caused by the periodic laser switching. When the laser turns off, heat dissipation occurs quickly, resulting in much lower heat accumulation during the heating phase compared to CW mode.

### 3.2. Evolving 2-D Strain Field Map

**Figure 5:**
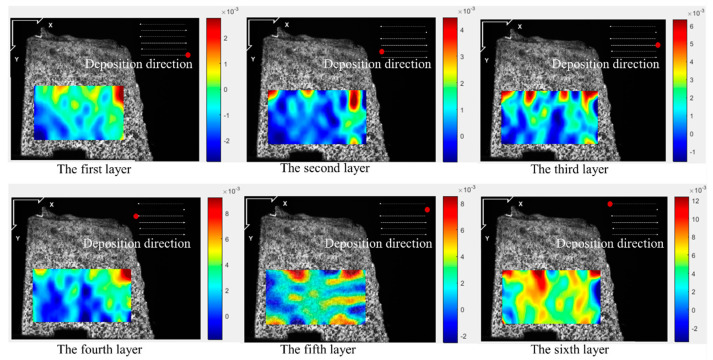
Two-dimensional longitudinal strain maps during the deposition stage (CW mode).

**Figure 6:**
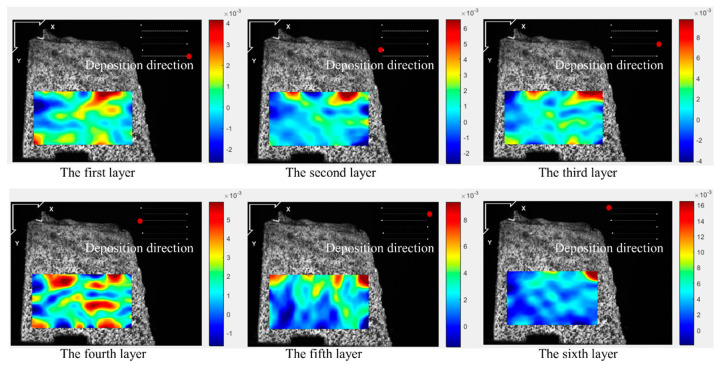
2-D vertical strain maps during the deposition stage (CW mode).

**Figure 7:**
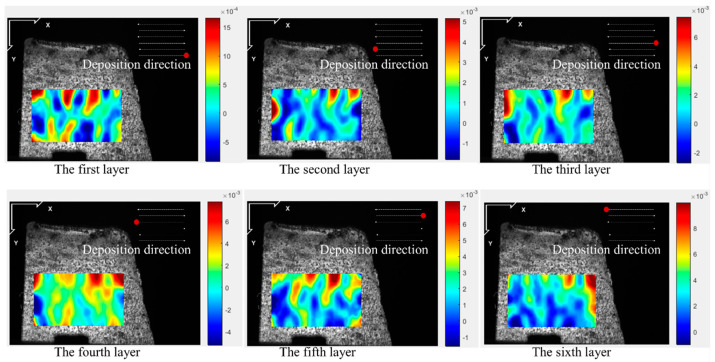
Two-dimensional longitudinal strain maps during the deposition stage (PW mode).

**Figure 8:**
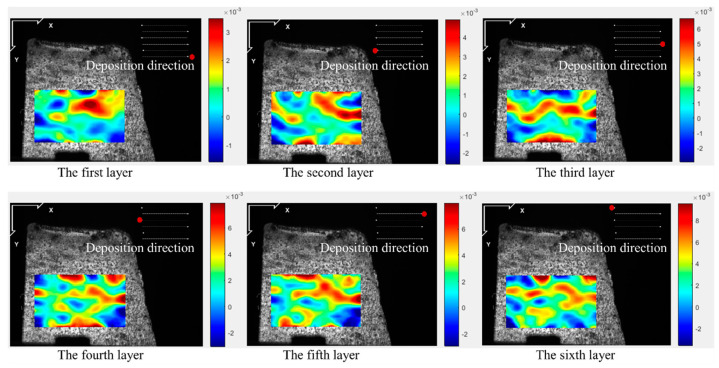
Two-dimensional vertical strain maps during the deposition stage (PW mode).

Figure 5, Figure 6, Figure 7 and Figure 8 illustrate the 2-D maps showing the evolution of longitudinal and vertical strain fields below the deposition layer during the six-layer deposition process for both CW and PW modes. The maximum strain areas (highlighted in red) appear above the visible strain cloud, with tensile and compressive strains (represented in purple and blue) distributed at the bottom to balance the overall strain state across the free surface. In the Y direction, a “tension-compression-tension” strain pattern is observed, whereas in the X direction, the strain distribution is irregular.

This irregularity in the X direction arises because the top of the strain cloud is closest to the molten pool, allowing ample heat conduction to the visualization area during the heating phase. As heat from the source reaches the area of interest, the metal expands due to heating, generating tensile strain near the heat source in the visualization area. Meanwhile, compressive strain develops further away from the heat source due to the constraints imposed by the underlying cooler metal. As the laser advances, the heat source moves, so the position of the strain cloud shifts with each layer’s deposition, resulting in the observed irregular strain pattern in the X direction.

**Figure 9:**
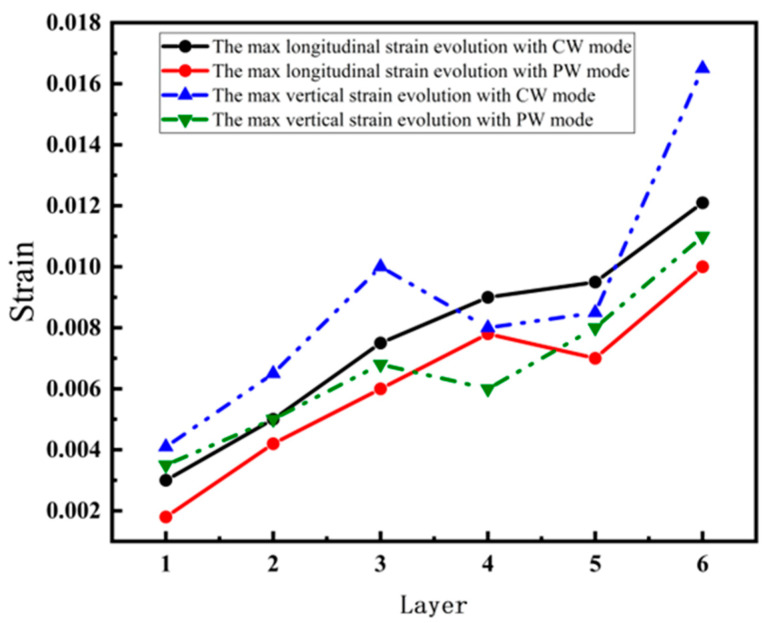
Two-dimensional maps of max strain during deposition stage (CW mode and PW mode).

The maximum longitudinal and vertical tensile strain value of each layer with both laser modes are extracted from Figure 5, Figure 6, Figure 7 and Figure 8, as shown in Figure 9. The strain increases by heat accumulation as the deposition layers grow. For CW mode, the maximum longitudinal and vertical strains increase from 2.9 × 10^−3^ and 4.0 × 10^−3^ in the first layer deposition to 1.2 × 10^−2^ and 1.7 × 10^−2^ in the sixth layer deposition, respectively. Meanwhile, the strain is relatively smaller for PW mode; the maximum longitudinal and vertical strains are 1.5 × 10^−3^ and 3.0 × 10^−3^ for the first layer, and correspondingly, 1.0 × 10^−2^ and 1.1 × 10^−2^ for the sixth layer. It can be seen that under the same laser power, compared with the CW laser mode, the highest transient longitudinal strain of the PW laser mode is reduced by 16.7%, and the highest transient vertical strain is reduced by 35.2%.

### 3.3. Residual Stress

**Figure 10:**
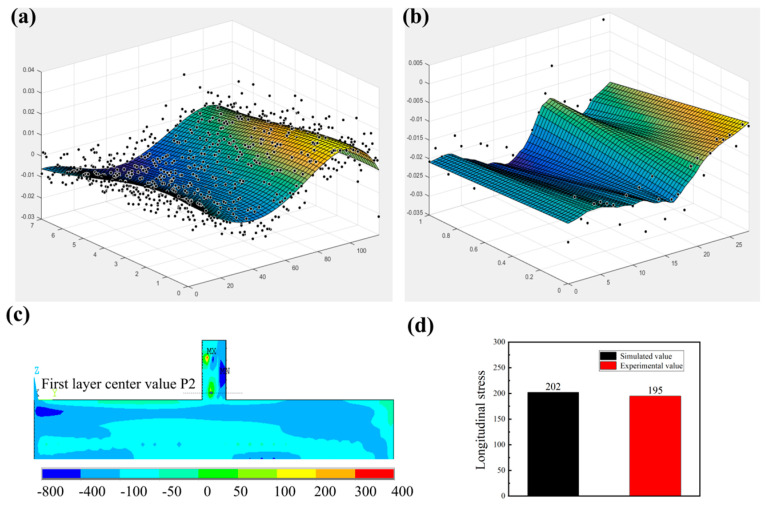
(**a**,**b**) The displacement measured in the encrypted and non-encrypted areas, respectively, caused by residual stress release; (**c**) 2-D mapping of the longitudinal residual stress ($\sigma_{x}$) in deposited sample; (**d**) Comparison of simulated values and experimental values at point P1.

Figure 10a,b show the displacement measured in both the encrypted and non-encrypted areas, respectively, due to residual stress release. Both areas display a U-shaped distribution, with raised edges and a concave center. The peak-to-trough displacement range is approximately 40 µm for the encrypted area and 35 µm for the non-encrypted area, which results from stress release caused by wire cutting, leading to deformation at the cutting surface. Notably, the sample edges also appear concave due to unavoidable data errors and measurement noise [23]. These errors and noise are filtered from the measured surface contour by fitting the data to a smooth analytical surface. After data processing, a 2-D map of residual stress for the 450 W sample, derived from the contour method, is shown in Figure 10c. The contour map in Figure 10c illustrates the longitudinal residual stress distribution within the deposited sample.

**Figure 11:**
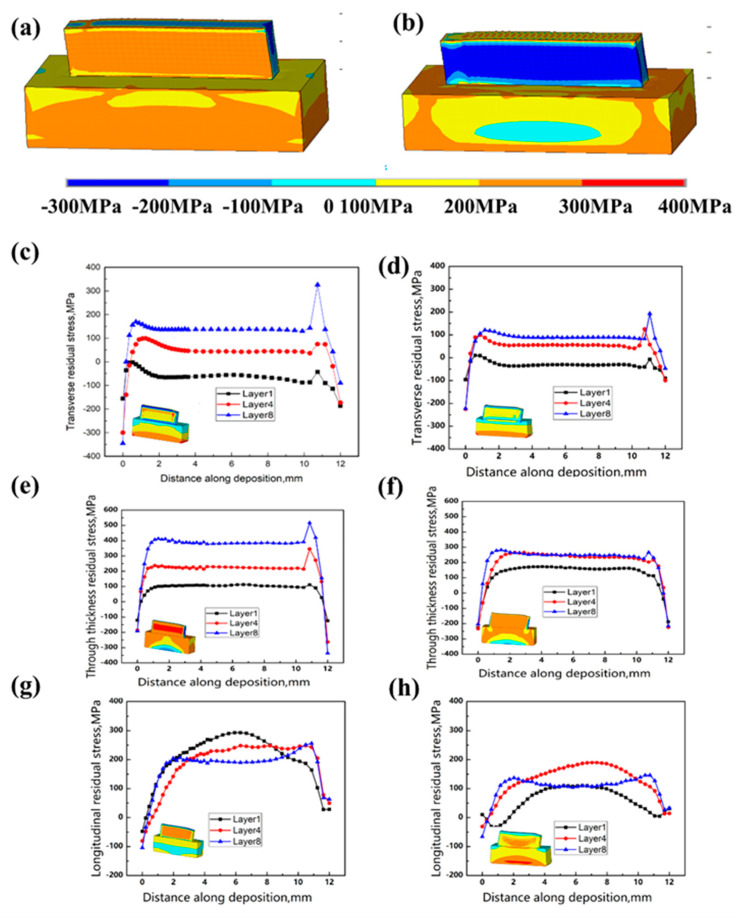
The longitudinal residual stress of the deposited sample along the midline of the cross section is distributed in the following directions: vertical, thickness, and longitudinal. (**a**) CW mode stress map, (**b**) PW mode stress map (**c**) Transverse stress along deposition direction for CW mode, (**d**) Transverse stress along deposition direction for PW mode, (**d**) Through thickness stress along deposition direction for CW mode, (**e**) Through thickness stress along deposition direction for PW mode, (**f**) Through thickness stress along deposition direction for PW mode, (**g**) Longitudinal stress along deposition direction for CW mode, (**h**) Longitudinal stress along deposition direction for PW mode.

A “tension-compression-tension” stress pattern is observed in the longitudinal residual stress for the CW mode along the height direction, which is a typical occurrence in conventional DED-LB deposited samples. Using the maps in Figure 10c, longitudinal residual stress data were extracted along the vertical mid-line of the cross section. Figure 10d shows the distribution of longitudinal residual stress along the longitudinal direction of the substrate. The simulated residual stress values at point P1 closely match those obtained experimentally, validating the accuracy of the simulated values. This model was then used to predict stresses in multiple directions, as illustrated in Figure 11.

Contour maps in Figure 11a,b depict the longitudinal residual stress distribution in the deposited sample block. Both samples are shown with scaled longitudinal residual stress cloud charts, accurately indicating the height and contour range. In CW mode, a “tension-compression-tension” pattern persists along the height, which is common in conventional DED-LB samples [22]. In PW mode, however, the longitudinal residual stress is tensile in the upper region but compressive at both the top and bottom. Changes in laser mode alter the molten pool size [21], which affects the depth profile of residual tensile stress. Consequently, the maximum residual tensile stress is slightly lower in PW mode compared to CW mode, with compressive stress forming on the free surface to counterbalance tensile stress. Due to the relatively low residual stress generated in PW mode, the stress distribution appears to be more uniform.

From the maps in Figure 11c,d, the transverse residual stress data along the deposition direction can be extracted at the midline of the cross section. The results indicate that switching the laser mode at the same power led to an approximate 40% reduction in maximum residual tensile stress, decreasing from 350 MPa to 210 MPa. Similarly, the thickness residual stress data along the deposition direction can be obtained from Figure 11e,f, also at the midline of the cross section. The thickness residual stress distribution along the deposition direction shows that changing the laser mode, while keeping the same power, resulted in a roughly 45% decrease in maximum residual tensile stress, from 550 MPa to 300 MPa. Figure 11g,h provide longitudinal residual stress data along the deposition direction, extracted along the midline of the cross section. These figures illustrate that altering the laser mode, with power unchanged, reduced the maximum residual tensile stress by approximately 32%, from 300 MPa to 202 MPa.

### 3.4. Mechanical Properties

**Figure 12:**
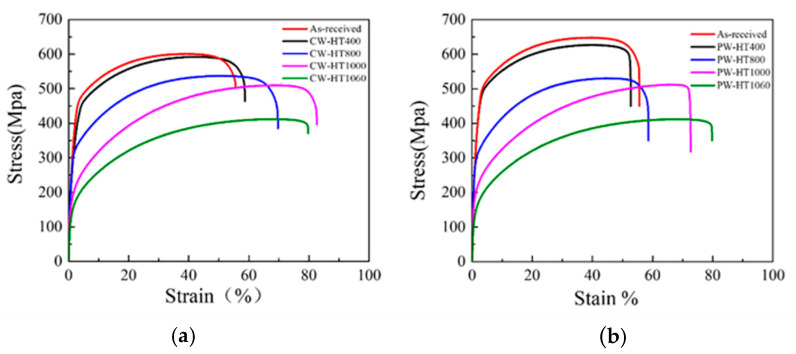
Tensile properties observed with CW and PW at different temperatures. (**a**) Mechanical properties of the samples after heat treatments under CW mode. (**b**) Mechanical properties of the samples after heat treatments under PW mode.

Figure 12a presents the tensile curves for DED-LB samples under CW mode after various degrees of annealing treatment. As the annealing temperature increased, the yield strength dropped from 460 MPa (as received) to 184 MPa (HT1060). The yield strength for each sample is listed in Table 3, with 160 MPa being the standard yield strength for 316 L stainless steel [24,25]. After HT1060 treatment, the yield strength exceeded that of typical 316 L steel. The ultimate tensile strength decreased from 607 MPa (as received) to 411 MPa (HT1060) as temperature increased, a trend commonly attributed to changes in DED-LB microstructures [24]. Figure 12b shows the tensile curves for DED-LB samples under PW mode following similar annealing treatments. Table 4 lists the yield strength for each sample, with values decreasing from 500 MPa (as received) to 160 MPa (HT1060). The ultimate tensile strength dropped from 658 MPa (as received) to 380 MPa (HT1060) with increased temperature. It is evident that PW mode maintains ductility similar to that of CW mode, but exhibits significantly improved strength. Under identical laser power, PW laser mode achieved a 10.8% increase in yield strength and an 8.3% increase in ultimate tensile strength compared to CW laser mode.

## 4. Discussion

### 4.1. Temperature Gradient Mechanism (TGM)

The primary factor influencing residual stress in a deposited sample during DED-LB is the temperature differential within the solid-state region. According to the temperature gradient mechanism (TGM) [28], when a material is heated in a specific area, a sharp temperature change occurs near the laser-induced heat source. The thermal strain generated in the solid material is strongly affected by this temperature differential. Higher temperature gradients lead to more pronounced thermal expansion and cooling-induced shrinkage, resulting in increased thermal strain in the affected area. After the deposited sample cools, residual stress levels rise, and the thermal strain *ε_th_* in the solid area can be mathematically expressed by Equation (6) [9]:

(6) $$\varepsilon_{th}=-\alpha (T-T_{0})=\varepsilon_{e}+\varepsilon_{p}+\varepsilon_{0}$$

T is the local temperature, $T_{0}$ is initial temperature, $\varepsilon_{e}$ is the elastic strain, $\varepsilon_{p}$ is the plastic strain, $\varepsilon_{0}$ includes the other inelastic strain such as that from phase transformation and creep, α is the coefficient of thermal expansion (CTE). Therefore, the value of the temperature gradient directly affects the magnitude of thermal strain, thus affecting the final residual stress.

Table 5 displays the greatest X-temperature gradient, maximum X-thermal strain, and maximum X-residual stress of the deposited sample during DED-LB at Point 1. The table indicates that the maximal thermal strain and average temperature gradient of the solid-state region in PW mode are approximately 16.7% and 16.7% lower than in CW mode, respectively. This is one of the reasons why the PW laser mode reduces residual stress.

### 4.2. Cyclic Thermal Stress and Crack Formation

**Figure 13:**
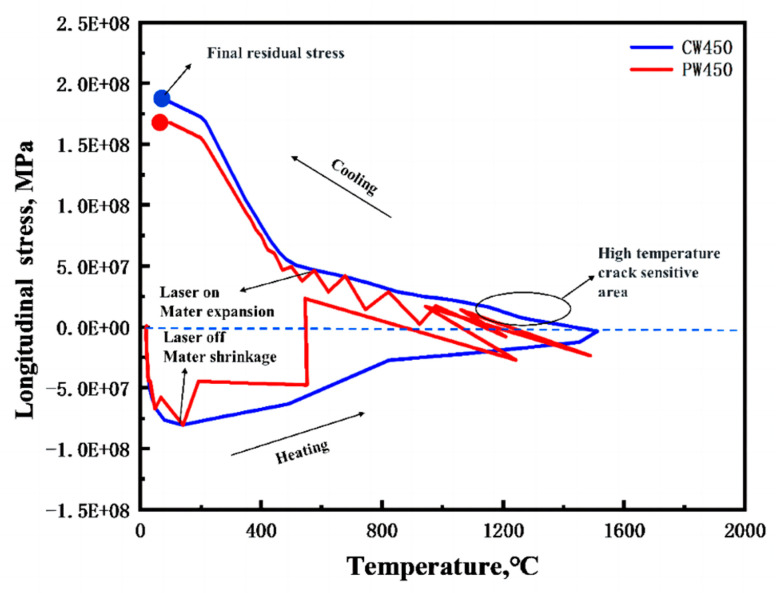
CW and PW mode temperature–longitudinal residual stress curves.

The relationship between temperature and stress was analyzed to further explore the mechanical deformation of stress components. Consequently, the temperature–stress relationship in both CW and PW modes was simulated using the commercial finite element software ANSYS 18.0. The results for Point A are schematically illustrated in Figure 13. During the heating process, the cladding metal experiences compressive stress within a specific temperature range. At the peak temperature of 1550 °C, the cladding metal stress reaches 0 MPa. Tensile stress begins to appear when the temperature reaches 1250 °C.

**Figure 14:**
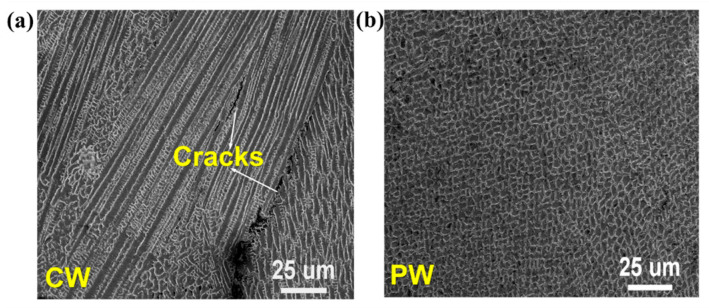
The microstructure of CW and PW mode samples (**a**) CW mode sample, (**b**) PW mode sample.

Another region of interest is the “brittleness temperature zone”, a high-temperature, crack-sensitive region where hot cracks may form if tensile stress on the cladding metal exceeds its plasticity. Upon cooling to room temperature, significant tensile strain arises as the laser energy dissipates, with residual tensile stress eventually reaching 190 MPa. Conversely, the temperature–stress relationship for the PW laser mode, represented by the red curve, shows similar oscillations in stress trends across all directions during heating. The thermal stress rises quickly and fluctuates significantly before cooling fully. Ultimately, residual stress increases continuously, reaching a final tensile stress of 160 MPa upon complete cooling. The temperature–stress relationship curve helps in understanding the crack development mechanism during cladding. As the CW mode’s temperature–stress curve passes through the “brittleness temperature zone”, numerous thermal cracks appear in the microstructure, as shown in Figure 14a. In contrast, by avoiding crack-sensitive regions during solidification, PW mode reduces hot crack formation, resulting in an almost crack-free microstructure, as depicted in Figure 14b.

### 4.3. Geometrically Necessary Dislocations (GNDs)

**Figure 15:**
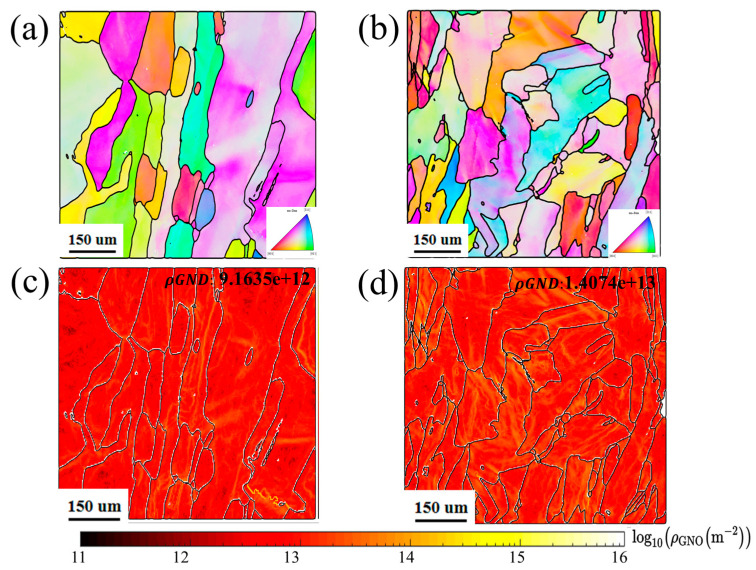
The microstructure of CW and PW modes may be described as follows: (**a**) The grain morphology of the CW mode, (**b**) The grain morphology of the PW mode, (**c**) The geometrically necessary dislocations (GNDs) of the CW mode, (**d**) The geometrically necessary dislocations (GNDs) of the PW mode.

The microstructure of 316 L stainless steel is characterized by grain size and geometric dislocation density, as shown in Figure 15. Figure 15a illustrates the particle morphology of a CW mode sample, revealing columnar dendrites growing opposite to the direction of heat transfer. The particles have an average diameter of 77.287 µm, a typical feature of additive manufacturing [29]. In Figure 15b, a substantial quantity of geometrically necessary dislocations (GNDs) is observed at the grain boundary, with an average GND value of approximately 9.1635 × 10^12^ m^−2^, as calculated from grain morphology. In the PW mode sample, grain morphology remains largely unchanged, but the grain size is reduced, ranging from 53.1 to 76.1 µm, as depicted in Figure 15c. In addition to a high concentration of GNDs at the grain boundaries, numerous dislocations are present within the grains, highlighted in yellow in Figure 15d. Consequently, the average GND value in the PW sample is 1.4074 × 10^13^ m^−2^, nearly double that of the CW sample. This high dislocation density is attributed to the thermal processing involved in DED-LB. Notably, samples with minimal residual stress in this study exhibit small grain sizes and a high density of dislocations.

This observation aligns with findings in the literature [30,31], and can be attributed to the pulse-wave laser’s ability to increase the cooling rate of the molten pool by an order of magnitude. The primary factor influencing grain size is the cooling rate; a higher cooling rate leads to smaller grains [32]. A larger number of grain boundaries indicates smaller grains, and under thermal stress, dislocations tend to accumulate and stack at these boundaries, resulting in increased GNDs [33]. The migration of dislocations driven by thermal stress due to temperature gradients leads to higher GNDs. In other words, the increase in GNDs corresponds to dislocation motion, which consumes thermal stress, consistent with previous studies [34,35].

The increase in GNDs reflects thermal stress relaxation. In PW mode, more thermal stress is dissipated, leading to lower residual stress. Additionally, as reported by T.R. Smith et al. [8], increased dislocation density contributes to higher yield strength, which enhances mechanical properties. This indirectly explains why, as discussed in Section 3.4, under the same laser power, PW laser mode achieved a 10.8% increase in yield strength compared to CW laser mode.

### 4.4. Phase Transition of Solids and Additional Potential Factors

**Figure 16:**
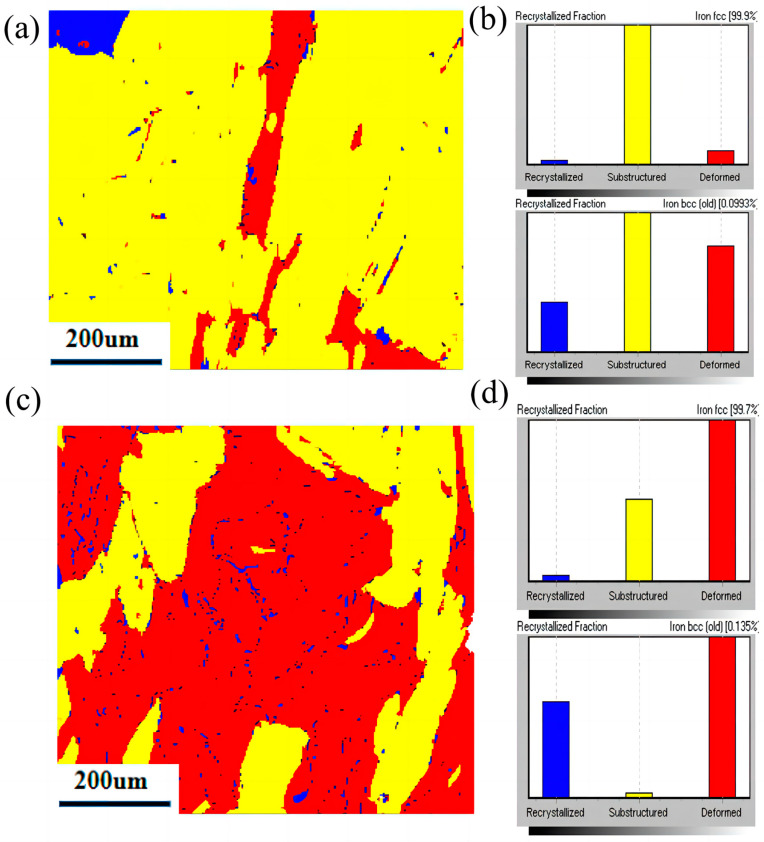
Recrystallization and phase diagram in CW mode and PW mode, (**a**) Recrystallization cloud image for CW mode, (**b**) Recrystallization fraction for CW mode, (**c**) Recrystallization cloud image for PW mode, (**d**) Recrystallization fraction for PW mode.

The influence of thermal history-induced phase transitions is another major factor affecting residual stress in the DED-LB process [36,37]. When cooling induces a partial transformation from FCC to BCC, compressive stress increases, and tension is relieved due to volumetric expansion [35,38]. In this analysis, Figure 16 displays the final solid phase distribution for both laser modes, based on EBSD data. Nearly 99% of the material remains in the FCC phase under both laser modes. However, the fraction of BCC phase in PW mode is 0.135% higher than in CW mode, with most BCC phases consisting of recrystallized grains, suggesting that recrystallization from solid phase change reduces residual stress in PW mode. During cooling, a partial transition from FCC to BCC contributes to additional compressive stresses and releases stress due to volume expansion [39]. This indicates that the observed reduction in residual stress in PW mode is not solely due to solid phase change.

In our previous study [40], we observed that solidified microstructures could trigger dynamic recrystallization under suitable temperature and thermal strain conditions induced by thermal cycling during direct energy deposition. The increased thermal cycling in pulsed laser mode compared to continuous laser mode may promote recrystallization nucleation, which could explain the greater toughness observed in pulsed mode compared to continuous laser mode.

**Figure 17:**
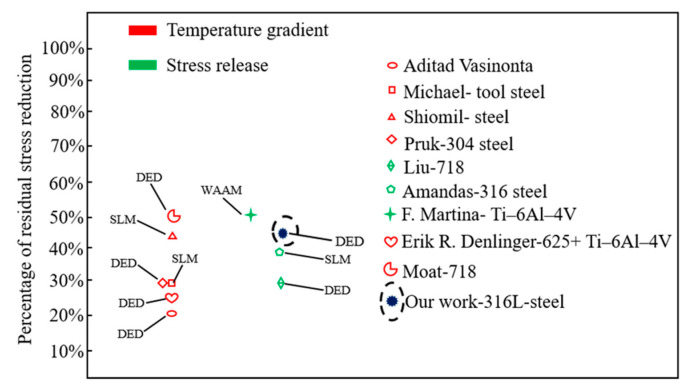
The impact of various methods and materials on residual stress reduction [41,42,43,44,45,46,47].

Figure 17 summarizes the effects of residual stress reduction achieved by various researchers in laser additive manufacturing, utilizing different techniques across various materials. Notably, the PW laser method employed in this study can reduce residual stress in the deposited sample by approximately 45%. This reduction is attributed to the PW laser’s ability not only to decrease the temperature gradient but also to induce compressive stress within the heat-affected zone. This outcome nearly doubles the effectiveness of other treatments that solely lower the temperature gradient to reduce residual stress. Compared to post-treatment and other stress relaxation methods, such as heat treatment or rolling, the PW laser method demonstrates a significant reduction impact. Moreover, the in situ residual stress reduction techniques proposed in this study have the potential to minimize post-treatment time, thereby enhancing manufacturing efficiency.

## 5. Conclusions

DIC experiment results show that, at the same laser power, the maximum transient longitudinal strain in PW laser mode is reduced by 16.7% compared to CW laser mode, while the maximum transient vertical strain is reduced by 35.2%.

The crack growth mechanism during cladding can be analyzed using the temperature–stress relationship curve. For samples produced in CW mode, this curve enters the “brittleness temperature zone”, which leads to the formation of numerous hot cracks. Conversely, PW mode samples are nearly crack-free, as the metal avoids crack-sensitive regions during solidification, reducing the likelihood of hot crack formation.

Periodic laser switching in PW mode induces compressive stress and results in a lower temperature gradient compared to CW mode. Both factors contribute to a reduction in residual stress and improved uniformity in the deposited samples, with PW mode achieving a residual stress reduction rate of approximately 45%. Additionally, while PW mode’s ductility is similar to that of CW mode, its strength is notably enhanced. Specifically, PW laser mode demonstrates a 10.8% increase in yield strength and an 8.3% increase in ultimate tensile strength relative to CW laser mode.

Thermal stress relaxation in PW mode is evidenced by higher GND values, as more thermal stress is dissipated, leading to reduced residual stress. Although solid-state phase changes can significantly decrease residual stress, PW mode does not induce such phase changes, and thus does not contribute to residual stress reduction in this aspect.

## Figures and Tables

**Table 1:** Element composition of 316 L stainless steel powder.

Element	Fe	Cr	Ni	Mo	Si	Mn	O	S	C	P
Percentage (%)	66.01	17.75	11.41	2.65	0.69	1.4	0.056	0.006	0.015	0.01

**Table 2:** Laser processing parameters.

Number	CW/PW	Power (W)	Scanning Speed (mm/s)	Pulse Frequency (HZ)	Duty Ratio
1	CW	450	6	/	/
2	PW	450	6	5	50%

**Table 3:** Mechanical properties of the samples after heat treatments under CW mode.

	As-Received	HT400	HT800	HT1000	HT1060	Cast [26,27]	Wrought [26]
**σy (MPa)**	460 ± 7	450 ± 5	325 ± 5	233 ± 7	184 ± 7	160	365
**σu (MPa)**	607 ± 4	608 ± 7	530 ± 2	509 ± 7	411 ± 5	450	555
**εt (%)**	39 ± 1	40 ± 1	45 ± 1	65 ± 1	72 ± 1	43	30

**Table 4:** Mechanical properties of the samples after heat treatments under PW mode.

	As-Received	HT400	HT800	HT1000	HT1060	Cast [26,27]	Wrought [26]
**σy (MPa)**	510 ± 7	500 ± 5	300 ± 5	230 ± 7	160 ± 7	160	365
**σu (MPa)**	658 ± 4	627 ± 7	510 ± 2	510 ± 7	380 ± 5	450	555
**εt (%)**	45 ± 1	42 ± 1	45 ± 1	68 ± 1	75 ± 1	43	30

**Table 5:** Temperature gradient, thermal strain, and residual stress of deposited CW and PW samples.

	Max Temperature Gradient (°C/mm)	Max Thermal Strain	Max Residual Stress (MPa)
CW	558.47	1.2%	300
PW	465.75	1.0%	202

## Data Availability

The data presented in this study are available on request from the corresponding author.
